# Male Sex Is Independently Associated with Faster Disability Accumulation in Relapse-Onset MS but Not in Primary Progressive MS

**DOI:** 10.1371/journal.pone.0122686

**Published:** 2015-06-05

**Authors:** Karen Ann Ribbons, Patrick McElduff, Cavit Boz, Maria Trojano, Guillermo Izquierdo, Pierre Duquette, Marc Girard, Francois Grand’Maison, Raymond Hupperts, Pierre Grammond, Celia Oreja-Guevara, Thor Petersen, Roberto Bergamaschi, Giorgio Giuliani, Michael Barnett, Vincent van Pesch, Maria-Pia Amato, Gerardo Iuliano, Marcela Fiol, Mark Slee, Freek Verheul, Edgardo Cristiano, Ricardo Fernandez-Bolanos, Maria-Laura Saladino, Maria Edite Rio, Jose Cabrera-Gomez, Helmut Butzkueven, Erik van Munster, Leontien Den Braber-Moerland, Daniele La Spitaleri, Alessandra Lugaresi, Vahid Shaygannejad, Orla Gray, Norma Deri, Raed Alroughani, Jeannette Lechner-Scott

**Affiliations:** 1 John Hunter Hospital, Newcastle, Australia; 2 University of Newcastle, Newcastle, Australia; 3 Karadeniz Technical University, Trabzon, Turkey; 4 University of Bari, Bari, Italy; 5 Hospital Universitario, Sevilla, Spain; 6 Hopital Notre Dame, Montreal, Canada; 7 Neuro Rive-Sud, Quebec, Canada; 8 Maaslandziekenhuis, Sittard, The Netherlands; 9 Hotel-Dieu de Levis, Quebec, Canada; 10 University Hospital La Paz, IdiPAZ, Madrid, Spain; 11 Kommunehospitalet, Arhus C, Denmark; 12 Neurological Institute IRCCS Mondino, Pavia, Italy; 13 Ospedale di Macerata, Macerata, Italy; 14 Brain and Mind Research Institute, Sydney, Australia; 15 Cliniques Universitaires Saint-Luc, Brussels, Belgium; 16 Department of Neurology, University of Florence, Florence, Italy; 17 Ospedali Riuniti di Salerno, Salerno, Italy; 18 FLENI, Buenos Aires, Argentina; 19 Flinders Medical Centre, Adelaide, Australia; 20 Groen Hart Ziekenhuis, Gouda, Netherlands; 21 Hospital Italiano, Buenos Aires, Argentina; 22 Hospital Universitario Virgen de Valme, Seville, Spain; 23 Craigavon Area Hospital, Craigavon, Northern Ireland; 24 Hospital S. João, Porto, Portugal; 25 Multiple Sclerosis Clinic, International Center of Neurological Restoration, Havana, Cuba; 26 Royal Melbourne Hospital, Melbourne, Australia; 27 Jeroen Bosch Ziekenhuis, Den Bosch, Netherlands; 28 Francicus Ziekenhuis, Roosendaal, Netherlands; 29 AORN San Giuseppe Moscati, Avellino, Italy; 30 Dept Neuroscience and Imaging—Univ "G. d'Annunzio", Chieti, Italy; 31 Al-Zahara Hopsital, Isfahan University of Medical Sciences, Isfahan, Iran; 32 Craigavon Area Hospital, Armagh, Northern Ireland; 33 Hospital Fernandez, Capital Federal, Argentina; 34 Amiri Hospital, Qurtoba, Kuwait; University of Düsseldorf, GERMANY

## Abstract

**Background:**

Multiple Sclerosis is more common in women than men and females have more relapses than men. In a large international cohort we have evaluated the effect of gender on disability accumulation and disease progression to determine if male MS patients have a worse clinical outcome than females.

**Methods:**

Using the MSBase Registry, data from 15,826 MS patients from 25 countries was analysed. Changes in the severity of MS (EDSS) were compared between sexes using a repeated measures analysis in generalised linear mixed models. Kaplan-Meier analysis was used to test for sex difference in the time to reach EDSS milestones 3 and 6 and the secondary progressive MS.

**Results:**

In relapse onset MS patients (n = 14,453), males progressed significantly faster in their EDSS than females (0.133 vs 0.112 per year, P<0.001,). Females had a reduced risk of secondary progressive MS (HR (95% CI) = 0.77 (0.67 to 0.90) P = 0.001). In primary progressive MS (n = 1,373), there was a significant increase in EDSS over time in males and females (P<0.001) but there was no significant sex effect on the annualized rate of EDSS change.

**Conclusion:**

Among registrants of MSBase, male relapse-onset patients accumulate disability faster than female patients. In contrast, the rate of disability accumulation between male and female patients with primary progressive MS is similar.

## Introduction

In multiple sclerosis (MS), the female to male ratio is as high as 3:1 [1,2]. However, while men have a lower risk of developing MS, many natural history and patient cohort studies have suggested that male sex is associated with a poorer clinical outcome in relapse-onset cohorts (RRMS, SPMS). In this form of MS, representing up to 85% of all MS cases, male patients are reported to have a more rapid accumulation of disability [3,4,5,6], reach disability milestones more rapidly than their female counterparts [7,8,9,10,11], display a more malignant form of disease [12] and have a poorer recovery after the initial disease relapse than females [13].

The rate at which patients reach the secondary progressive disease course (SPMS) is also potentially impacted by sex. Male sex has been associated with a faster time to progressive MS in some studies [7,14] but not others [15,16].

Primary progressive MS (PPMS) is a clinically distinct form of MS in which the accumulation of disability occurs in the absence of clinical relapses and patients experience a progressive course from disease onset [17,18]. PPMS patients are older at disease onset [19] than RRMS patients and there the sex ratio is 1:1 [20]. Male PPMS patients have been shown to accumulate disability more rapidly than their female counterparts even in the early stages of the disease [8]. Although no predictive effect of sex on the time to reach EDSS 6 has been seen [21, 22], sex has been shown to have prognostic value when it comes to disease progression to higher levels of disability with faster rates of reaching EDSS levels of 8 [23] and 10 [24] observed in male PPMS patients. In a multicentre prospective study of prognostic factors in PPMS, conducted across 5 European countries [25], male PPMS patients were also shown to be twice as likely to deteriorate than female patients over 10 years of follow up.

In the current study, we have used longitudinal clinical outcome data, extracted from a large international, multicentre database, the MSBase Registry [26], to assess if sex affects the accumulation of disability in relapsing remitting MS, progression to the secondary progressive phase and disease progression in the primary progressive MS phenotype.

## Methods

### The MSBase Registry

The MSBase Registry was established in 2006[26] and is a prospective, international, web-based database collecting standardized clinical outcomes in MS patients using an agreed minimum dataset. The Registry collects clinic-based and private practice based information on people with MS. From its inception in 2006, data was prospectively collected from patients and entered on to the Registry together with retrospective data derived from the patient’s medical history thereby enabling long term clinical follow up. Patient information is recorded at each of the collaborating centers using the offline medical record iMED and then uploaded to the MSBase web portal. The use of MSBase as a research platform was approved by the Melbourne Health Human Research Ethics Committee and by the local human research ethics committee at all participating centers, or in some cases relevant exemptions were granted, in accordance with local laws and regulations. Signed informed consent was obtained from each participating patient if required.

Quality assurance of clinical data was maintained by inbuilt data quality checking in the iMED local record system and data quality reporting from MSBase. To ensure consistency of EDSS evaluations all neurologists completed the Neurostatus certification (Kappos L, http://www.neurostatus.net) or provided evidence of prior completion of this certification.

### Statistical Methods

#### Change in EDSS over time

The analysis of change in scores on the EDSS was done separately for those with PPMS and for the combined group of patients with SPMS or RRMS.

In each cohort outcome measurements included evaluations of the impact of sex on disability accumulation by evaluating the effect on annualized EDSS change.

All statistical analyses were performed using SAS 9.3 statistical software program (SAS Institute, Cary, NC, USA).

A repeated measures analysis, using data from all EDSS time points, after initial EDSS, recorded at the first visit recorded to the MSBase Registry, was conducted using generalised linear mixed models. The outcome in the model was the subjects’ EDSS. The main independent variables were sex, years since onset of MS and the interaction term of sex by years since disease onset. The p-value from the interaction term was used to determine if there was a statistically significant difference between men and women in the change in EDSS scores over time. The time variable in the model was calculated as date of visit (for assessment of EDSS) minus the date of MS symptom onset. The models were adjusted for a number of potential confounding variables including treatment (the numbers of days used prior to the last EDSS assessment), age at first symptom and country of birth. Available treatments during the observation period of this study included Interferon β-1a formulations, Interferon- β1b, glatiramer acetate and natalizumab.

#### Time to reach EDSS milestones 3 and 6

The time to reach EDSS milestones 3 or 6 were estimated for each sex in the RR/SPMS and PPMS cohorts, used in the evaluations of the change in EDSS over time, by Kaplan Meier Survival models. The time period to reach EDSS 3 or 6 was evaluated from the time of onset of symptoms to the time of the first clinic visit recorded to the Registry at which the EDSS was above the cut point of EDSS 3 or EDSS 6. The statistical models used assumed that all patients used in the analysis had an EDSS less than 3 or 6 at the time of onset of symptoms, however, the EDSS milestones may have been reached at the first clinic visit entered to the Registry.

#### Time to progression to SPMS

The time of SPMS disease course conversion from RRMS was derived from the time of onset of symptoms to the onset of SPMS diagnosis provided by the participating neurologist at each participating centre. The diagnosis of SPMS was performed according to the international accepted definition of demonstrated disease progression independent of relapses for at least 6 months in patients previously classified as RRMS [27]. A Cox Proportional Hazards model was used to examine time from onset of symptoms to diagnosis of SPMS among patients. This model was also adjusted for treatment, age at first symptom and country of birth.

## Results

MS patients were enrolled to the MSBase Registry if their clinic data met the minimum dataset requirements for eligibility which included the collection of mandatory patient profile data at the entry visit, and at least an annual follow-up visit. Key data collected from each visit included relapse rate, relapse treatment, MS specific therapies used, EDSS and diagnostic tests performed [26]. On February 6 2012, data was extracted from the MSBase Registry for 18,885 MS patients who fulfilled MSBase Registry minimum dataset requirements. Data was derived from 55 collaborating centers in 25 countries. Patients were considered evaluable in assessments of the change in disability accumulation over time, in the current study, if there was a recorded date for the confirmed diagnosis of MS and of their MS course designation as being either RRMS, SPMS or PPMS. Dates were also required for the visit dates during which their EDSS scores were assessed. When applying these criteria, for evaluable data required for this study, we derived data for 14,453 MS patients, with a diagnosis of RRMS or SPMS, and 1373 patients with a diagnosis of PPMS. Patients were considered evaluable for Kaplan Meier analysis, of the time to developing SPMS, if there was a recorded date for the onset of disease symptoms and they were not progressive from onset. When applying these criteria we derived data from 16,487 MS patients for survival curve analysis.

The sex distribution in combined RR/SPMS patient group showed a female to male ratio of 2.5:1. Demographic features of each sex were comparable at the initial onset of MS, however statistically, males were significantly older than females and their initial EDSS was higher than that of their female counterparts (Table 1). The time period between their initial onset of symptoms to their most recent visit date for both males and female patients was comparable, with a median of 9.9 years (min 2 days, max 34.96 years) for males and a median of 10.4 years (min 1 day, max 34.99 years) for females. 70% of of all patients were exposed to disease modifying therapies, during the observational period and this occurred to the same extent in both male and female populations.

**Table 1:** Demographics of patients with relapsing- remitting (RR) / secondary progressive (SP) and primary progressive (PP) MS at the initial visit recorded to the MSBase Registry.

	Variable	Males	Females	p-value
**RR/SP MS**	**Number**	4051	10304	
	**Age at onset (years)**	30.5 (9.8)	30.0 (9.8)	0.011
	**Initial EDSS**	2.8 (1.9)	2.6 (1.9)	0.001
**PP MS**	**Number**	619	747	
	**Age at onset (years)**	39.7 (10.6)	40.1 (11.0)	0.517
	**Initial EDSS**	4.6 (5.1)	5.1 (5.5)	0.318

Data are shown as mean (+/- SD)

Unlike the marked female sex bias noted in the RR/SP MS group, the primary progressive group reflected a more even distribution between sexes, with a female to male ratio of 1.2:1 (Table 1). Diagnosis of primary progressive MS occurred in an older patient population with the age at disease onset being approximately 10 years later than that seen in the RR/SPMS group (Table 1). The mean level of disability recorded at the initial visit entered on the Registry was higher in the PPMS group (EDSS 4.7 ± 1.9) compared to that seen in RR/SP MS patients (EDSS 2.8±1.9) and was comparable between sexes (Table 1).

**Fig 1:**
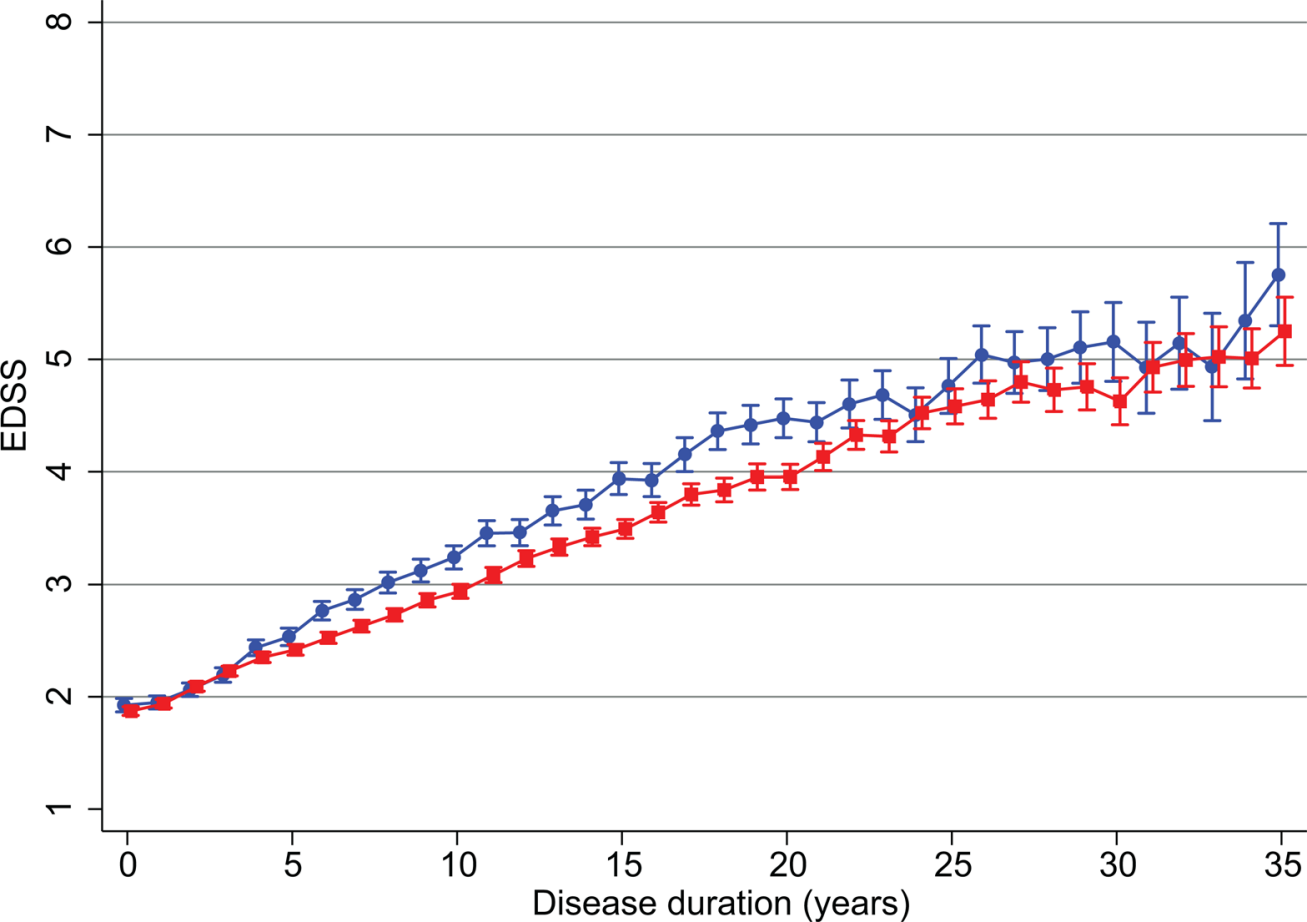
Mean value of EDSS for men and women with secondary progressive or relapsing remitting MS by number of years since initial EDSS. EDSS is shown as mean value ± SD. Disease duration was derived as the date of the visit at which the EDSS was determined minus the date of onset of MS symptoms. Red squares = females, blue circles = males.

In the RR/SP MS patient group the effect of sex on the rate of change in disability up to a maximum of 35 years from the initial onset of symptoms to the most recent recorded visit date was evaluated (Fig 1). Table 2 shows the number of patients contributing to this evaluation at 5 year intervals between visits. There was a statistically significant increase of 0.124 (0.119 to 0.129) units per year in the mean level of EDSS scores over time for men (P<0.001) and 0.107 (0.104 to 0.111) for women (P<0.001). The increase in the mean level of EDSS differed significantly between sexes (P<0.001) with the disability increasing slightly faster in males than females (Fig 1). The number of valid EDSS measurements per person contributing to this evaluation was comparable between males (median 5, min 1, max 68) and females (median 6, min 1, max 73).

**Table 2:** Number of male and female RRMS/SPMS patients who provided at least one valid EDSS measurement in each 5 year period.

Years since initial symptoms	Male	Female	Total
**<4**	2302	5561	7863
**5 to 9**	1951	4953	6904
**10 to 14**	1433	3696	5129
**15 to 19**	859	2301	3160
**20 to 24**	540	1408	1948
**25 to 29**	307	810	1117
**30 to 34**	151	479	630

The time to reach disability milestones 3 and 6 was evaluated in 14,355 RR/SPMS patients. On average it took 8 years after the initial onset of symptoms for the male RR/SP MS population to reach EDSS 3 while it took 10 years for the female population to reach this level. At the higher level of disability in the male population, EDSS of 6 was reached at 32 years after the initial visit, whilst the mean for the female population did not reach this level of disability within the observed time frame (Fig 1).

**Fig 2:**
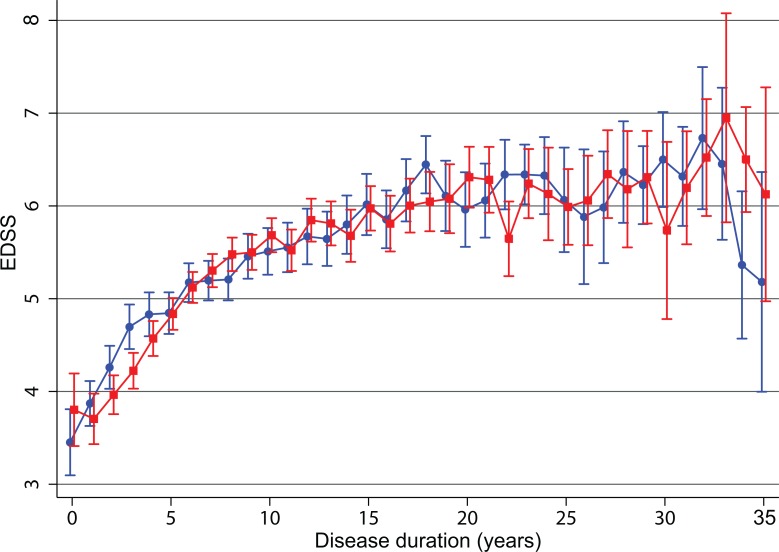
Mean value of EDSS for men and women with primary progressive MS by number of years since initial EDSS. EDSS is shown as mean value ± SD. Disease duration was derived as the date of the visit at which the EDSS was determined minus the date of onset of MS symptoms. Red squares = females, blue circles = males.

In the PPMS population, there was a statistically significant increase in EDSS scores over time for men (P<0.001) and for women (P<0.001) up to 35 years following the initial onset of symptoms, but unlike the sex differences seen in the RR/SP group, there was no significant difference in the annualized change in EDSS between sexes in PPMS (P = 0.889) (Fig 2). Table 3 shows the number of patients contributing to this evaluation at 5 year intervals between visits. The median duration between the initial onset of symptoms and the most recent visit date recorded to the Registry was comparable between males (median 10.57 years, min 0.23 years, max 34.88 years) and females (median 11.28 years, min 0.42 years, max 34.90 years). The number of valid EDSS measurements per person contributing to this evaluation was comparable between males (median 4, min 1, max 63) and females (median 4, min 1, max 60).

**Table 3:** Number of males and female PPMS patients who provided at least one valid EDSS measurement in each 5 year period.

Years since initial symptoms	Male	Female	Total
**<4**	268	312	580
**5 to 9**	301	374	675
**10 to 14**	211	283	494
**15 to 19**	132	192	324
**20 to 24**	84	103	187
**25 to 29**	49	56	105
**30 to 34**	28	33	61

The EDSS level at onset of symptoms in the PPMS group (n = 1366) was higher than that seen in the RRMS group (Table 1) and the increase in disability was faster: it took approximately 15 years after the onset of symptoms to reach a mean EDSS 6 (Fig 2).

**Fig 3:**
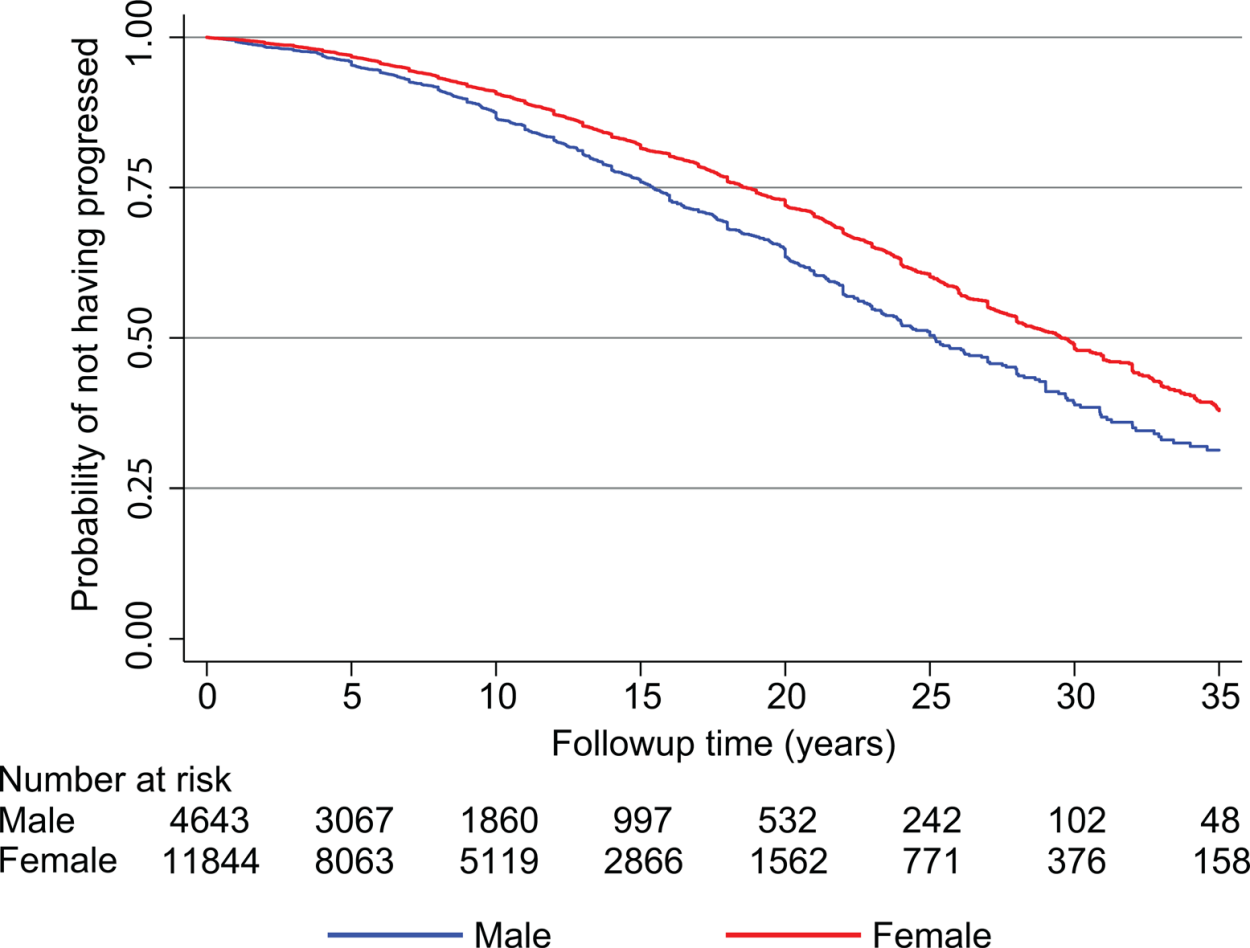
Kaplan-Meier estimates of time to secondary progressive MS by sex.

Disease progression rate was evaluated from the onset of symptoms to the time to reach SPMS using Kaplan-Meier survival analysis (Fig 3). A diagnosis of SPMS was recorded in 2,664 MS patients. The derived median time to reach secondary progressive MS was 25.1 years for men and 29.5 years for women. Survival analysis, using Cox Proportional Hazards models, suggest that women had a statistically significant reduced risk of developing SPMS compared to men [HR (95% CI) = 0.77 (0.67 to 0.90); P = 0.001], indicating that in this multinational MS cohort study, male RRMS patients reach the progressive phase earlier than females.

## Discussion

In this study we evaluated the effect of sex on the rate of accumulation of disability and time to disease progression in 15,721 MS patients with a confirmed diagnosis of multiple sclerosis. This represents the largest study population evaluated to date and exceeds by far regional MS natural history studies conducted in Lorraine (2,871 cases, [4,5]), Lyon (1,844 cases, [7,8]), and Rennes, France (2,054 cases, [10]), or London Ontario (1,099 cases [28]) and British Columbia, Canada (2,387 cases, [29]). Data derived from the MSBase database mirrors the sex bias towards female patients in the relapsing remitting and secondary progressive MS phases reported in other large regional epidemiological studies [2,3,4,6,7,8,10,13]. The rate of disease progression we observed is also comparable to that seen in regional cohort studies with the time taken to reach early disability milestone of EDSS 3 being similar to that seen in Rennes, France (10 years, n = 1609) [10], and London Ontario, Canada (8 years, n = 2236) [28]. With respect to reaching a higher level of disability (EDSS 6) our male cohort reached this level 32 years following their initial recorded EDSS while our female cohort did not reach this level of disability within the maximum observation period of 34 years. This represents a slower rate of progression in female patients than reported by others, with female cohorts in French databases from Lorraine, Lyon and Rennes reaching EDSS 6 within 24 years, 23 years and 20 years, respectively [4,7,8,10]. The most rapid rate of disability progression was reported in Canada with the total London Ontario RRMS patient population reporting a median time to reach EDSS 6 within 15 years [28] while the Canadian British Columbia cohort displayed a far more protracted rate of decline with a median time to EDSS 6 of 27 years [29]. There are several possible factors that may account for the regional variations in the rate of accumulation of disability. The clinical outcomes data we derived from the MSBase Registry was analyzed a decade after several of the French datasets were acquired. Consequently, it is possible that the range of disease modifying therapies recorded in the MSBase Registry reflects current clinical practices they may be impacting on clinical outcomes which is in contrast to earlier studies performed at a time of a much more limited treatment armory [7,8]. This may also be contributing to a shift in the severity of cases presenting to clinics with a milder disease course that could be impacting on the clinical outcome profile we are recording to the MSBase Registry.

In relapse-onset MS patients we have shown that male patients start off with a slightly higher EDSS than female patients at their first clinic visit entered to the MSBase Registry and they accumulate disability faster. This finding has been supported by others who have shown a more rapid attainment of EDSS scores of 4, 6 and 7 from onset in male patients [3,6,8,10]. However, the effect of sex on rate of disability accumulation in relapsing remitting MS has been challenged, with the suggestion that it is in fact a two stage process, and factors such as sex only impacting on the attainment of disability levels up to EDSS 3 [8,10], and not on the level of disease accumulation in the later phase, from EDSS 3 up to EDSS 6. This suggests that the sex effect on disability change was confined to the early inflammatory disease process and has limited influence on the later, degenerative phenotype. While we did not explore these possibilities, we do show that annualized EDSS change was faster in males across a range of EDSS levels and a more rapid attainment of EDSS 6 was seen in male patients.

There is consensus between regional cohort studies regarding the time taken for relapsing remitting patients to reach the secondary progressive disease phase with most studies reporting a 20 year duration from disease onset to onset of secondary progressive MS [4,30,31]. A comparable time course for the development of progressive disease has also been reported by others in their female populations exclusively [4,14,31]. In contrast, the median time to reach this phase was delayed in our MSBase patient population up to 29.5 years for female patients. Our findings increase the weight of evidence for a significant sex effect on the time to SPMS and supports other regional studies that that show a more rapid rate of progression in males [4,9,14]. The exception being a study from Groningen, Netherlands [16] that was unable to demonstrate any impact of sex on disease progression.

One potential limitation of the current study was that the diagnostic criteria for reaching the secondary progressive phase was not dictated by MSBase, but rather determined by the treating neurologists at each participating site and the usage of the appropriate definition of SPMS was not audited. However, the international accepted definition was known to be implemented in clinical practice by a large number of MSBase investigators and it is unlikely that the criteria used differed between males and females and therefore do not explain our gender difference. It has also been suggested [32] that the clinical designation of SPMS is more accurate than deriving the time for reaching the progressive phase by applying an algorithm to clinical outcome data.

Baseline demographic data, derived from the first clinic visit entered to the Registry, in the current study also indicated that males were marginally but significantly older (mean 0.5 years) at relapsing remitting MS onset. Indeed the age at onset of symptoms has been proposed as a major prognostic factor of progression of disease, with an older age at disease onset associated with a poorer disease outcome [1,13], and a more rapid development of the progressive phase of the disease [15,16,33]. However, we still found faster disability progression in males after adjusting for age of onset differences.

Another potential variable that could impact on disease severity and progression in relapse remitting MS is relapse rate. It is, however, unlikely that the sex effects seen in our study are explained by relapse rate as recent evaluation of relapse incidence, utilizing clinical outcomes from the same MSBase registry [34], indicates that relapse frequency was higher in females compared to males, at all relapsing-onset disease stages. This suggests that other factors are contributing to the poorer clinical outcomes we are observing in male patients. Indeed, it has been suggested that factors other than relapse rate impact on long term disability in MS, evidenced by a lack of effect of relapse rate on long term disability accumulation or the time to reach the secondary progressive phase of MS [35,36].

Primary progressive MS is clinically distinct from relapsing-onset MS as it is believed to be associated with a prominent degenerative pathology from disease onset [17,20,27]. We evaluated clinical outcomes of 1336 primary progressive patients representing the largest clinical analysis of primary progressive MS conducted to date. The later age at onset of primary progressive MS patients and sex parity observed in our study population is comparable to that seen in regional cohort studies of primary progressive MS [20,21,23,28,35,37]. The time taken to reach EDSS 6 was almost halved compared to patients with relapsing remitting disease, suggesting a significantly poorer prognosis and more rapid rate of disability accumulation in this form of MS. The median time for primary progressive MS patients to reach the EDSS 6 milestone in the MSBase study population (15 years) is comparable to that seen in the British Columbia database (14 years, n = 552 [21]), but is more protracted than that reported in Wales (9.6 years, n = 234 [23], London Ontario (8.5 years, n = 216 [37]) and in Lyon (7.1 years, n = 282 [8]).

In the MSBase population there was no evidence of an effect of sex on the rate of accumulation of disability over time in primary progressive patients. There is an apparent lack of consensus on the prognostic value of sex on disease progression in primary progressive MS, which may in part be due to differences in the stages of disability accumulation and outcomes being evaluated. Male sex has been associated with a faster time to reaching EDSS milestones 4, 6 and 7 in the Lyon population [8], but not in the London Ontario group, where sex failed to impact on time to earlier EDSS milestones but EDSS 10 (death) was reached faster by male patients [37]. In a 10 year follow up study of 145 PPMS patients across 5 European centers, males with PPMS had worse outcomes [25]. However, our much larger study refutes this, finding no sex-associated difference in PPMS progression rates.

## Conclusions

We have shown that male MS patients with relapse-onset have more rapid annualized EDSS progression, shorter time to SPMS, and shorter time to EDSS milestones 3 and 6. On the other hand, in our large cohort of primary progressive MS patients, we found no sex-associated effects on rate of disability accumulation. Overall, our large cohort study showed a slower disease progression than previous studies, likely due to greater uptake of disease modifying drug treatment and the advent of more effective drugs in our cohort, although an increased ascertainment of milder MS cases could also contribute.
